# Genoprotective Properties and Metabolites of β-Glucan-Rich Edible Mushrooms Following Their In Vitro Fermentation by Human Faecal Microbiota

**DOI:** 10.3390/molecules25153554

**Published:** 2020-08-04

**Authors:** Athina Boulaka, Paraschos Christodoulou, Marigoula Vlassopoulou, Georgios Koutrotsios, Georgios Bekiaris, Georgios I. Zervakis, Evdokia K. Mitsou, Georgia Saxami, Adamantini Kyriacou, Maria Zervou, Panagiotis Georgiadis, Vasiliki Pletsa

**Affiliations:** 1 Institute of Chemical Biology, National Hellenic Research Foundation, 11634 Athens, Greece; aboulaka@eie.gr (A.B.); pchristodoulou@eie.gr (P.C.); mvlassopoulou@eie.gr (M.V.); mzervou@eie.gr (M.Z.); 2 Department of Nutrition and Dietetics, Harokopio University, 17676 Kallithea, Greece; emitsou@hua.gr (E.K.M.); gsaxami@hua.gr (G.S.); kyriacou@hua.gr (A.K.); 3 Laboratory of General and Agricultural Microbiology, Department of Crop Science, Agricultural University of Athens, 11855 Athens, Greece; georgioskoutrotsios@gmail.com (G.K.); giorgosbekiaris@yahoo.gr (G.B.); zervakis@aua.gr (G.I.Z.)

**Keywords:** edible mushrooms, β-glucans, faecal microbiota, in vitro fermentation, genoprotection, NMR-based metabolomics

## Abstract

A variety of bioactive compounds, constituents of edible mushrooms, in particular β-glucans, i.e., a group of β-d-glucose polysaccharides abundant in the fungal cell walls, have been linked to immunomodulating, anticancer and prebiotic activities. The aim of the study was the investigation of the genoprotective effects of edible mushrooms produced by *Pleurotus eryngii*, *Pleurotus ostreatus* and *Cyclocybe cylindracea* (Basidiomycota). Mushrooms from selected strains of the species mentioned above were fermented in vitro using faecal inocula from healthy volunteers. The cytotoxic and anti-genotoxic properties of the fermentation supernatants (FSs) were investigated in Caco-2 human colon adenocarcinoma cells. The FSs were cytotoxic in a dose-dependent manner. Non-cytotoxic concentrations were used for the genotoxicity studies, which revealed that mushrooms’ FSs have the ability to protect Caco-2 cells against tert-butyl hydroperoxide (*t*-BOOH), a known genotoxic agent. Their global metabolic profiling was assessed by ^1^H-NMR spectroscopy. A total of 37 metabolites were identified with the use of two-dimensional (2D) homo- and hetero-nuclear NMR experiments. Multivariate data analysis monitored the metabolic variability of gut microbiota and probed to biomarkers potentially associated with the health-promoting effects of edible mushrooms.

## 1. Introduction

Since the beginning of the current century, there has been an increasing interest in the exploitation of natural products to alleviate or reduce the risks associated with multifactorial diseases, namely, cardiovascular disease, diabetes, neurodegeneration and cancer [1,2]. Growing experimental evidence supports the significant role of bioactive polysaccharides in the aforementioned health-promoting properties. In nature, polysaccharides can be found in almost all living organisms, including tissues of seeds, stems and leaves of herbal plants, body fluids of animals, cell walls and extracellular fluids of bacteria, yeast and fungi, ranging in structure from linear to highly branched [3].

Many mushroom species are well-reputed for their health-promoting properties; however, their content in functional (and/or pharmaceutical) compounds and the respective mechanisms of biological activity have been studied only recently, revealing the crucial role of several fungal polysaccharides in anticancer therapy, stimulation of the immune system, as well as their prophylactic activity against oxidative stress, chemo/radio-therapy, pathogens and their potential for regulating and preventing hyperglycaemia and hypercholesterolemia [1,3]. Bioactive fungal polysaccharides seem to act as biological response modifiers due to their immunomodulatory activities, and even their anticancer properties have been linked to immunomodulatory effects rather than direct cytotoxicity [4,5]. They comprise a large group of biopolymers which are either part of the cell wall or may form intracellular inclusions and serve as energy reserves or are excreted extracellularly, providing a mechanism for cell protection or attachment to other surfaces. Many of them derive either from edible mushrooms or from GRAS (Generally Recognised as Safe) organisms, e.g., bakers’ yeast [3]. All these properties, along with the absence of toxicity, render the fungal polysaccharides ideal compounds for the development of novel functional foods or nutraceuticals to meet the consumers’ demand for healthier food.

In edible mushrooms, β-(1,3)-d-glucans and β-(1,3) (1,6)-d-glucans, in particular, constitute a large group of polysaccharides, essential constituents of the fungal cell wall that differ widely in the ratio and arrangement of the 1,3-and 1,6-β-glycoside bonds [6]. B-glucans have been extensively examined over the last decade, and various studies have supported the relationship between the molecular structure of β-glucan and its functionality. The structural characteristics of β-glucans, including specific glycosidic linkages, monosaccharide compositions, molecular weight and chain conformation, seem to affect their physiochemical and biological properties [7].

Due to their beneficial properties, β-glucans have been not only widely used in food but also in medical, pharmaceutical and cosmetic applications [2,3]. They are also present in cereal bran such as oat and barley and commonly produced as agricultural by-products due to their economic and environmental benefits [8]. Given their health benefits, the U.S. Food and Drug Administration (FDA) [9] has allowed the health claim on a food label for reduction of the cholesterol level when cereal β-glucan (0.75 g per serving) is included. Similarly, the European Food Safety Authority (EFSA) authorised a health claim related to the maintenance of normal blood cholesterol concentrations for soluble cereal fibres, particularly β-glucans from oat and barley [10]. Consequently, food manufacturers have made efforts to produce β-glucan-enriched substances from various cereal [8] and other sources [11,12] in order to incorporate them into a variety of food products. Since fungal polysaccharides have been reported to be effective immunomodulators, due to their structure, recent studies focused on exploiting yeasts and mushrooms’ β-glucans for the development of functional foods [13].

When ingested through food, β-glucans end up in the colon, where they are completely fermented by the intestinal microbiota, often increasing the populations of beneficial bacteria, such as those of the genera *Bifidobacterium* and *Lactobacillus*. They are, thus, considered potential prebiotics [14]. The gut microbiota (GM) is comprised of microbial communities colonising the human intestinal tract, creating an extremely dense ecosystem playing an important role in maintaining host organism homeostasis through a variety of mechanisms that have not yet been elucidated. The intestinal microbiota interact with the diet and the host’s epithelium and immune system, provide protection against invasion of pathogens, participate in the regulation of various metabolic pathways and ultimately induce significant alterations in the host’s physiology [15]. The link between the anticancer and immunomodulatory properties of mushrooms (and β-glucans) with their possible prebiotic activity on gut micro-organisms remains a central question and has been the subject of intense research over the last decade [16]. There is also evidence supporting the genoprotective effects of β-glucans in vitro [17,18,19], in vivo [20,21,22] and in polypectomised patients [23]. Nevertheless, the genoprotective properties of fungal polysaccharides are frequently attributed to their action as radical scavengers and not to their interaction with gut microbiota. To our knowledge, only a handful of studies address their potential genoprotective properties within the frame of their fermentation by intestinal microbiota, and the results indicate that further research is needed [24,25,26,27,28]. The human clinical trials conducted so far with isolated fungal polysaccharides or whole mushrooms of high β-glucan content are also very few and inconclusive [29]. Moreover, the question as to whether the metabolites produced, following their fermentation by intestinal microbiota, are responsible for the health-promoting properties of edible mushrooms remains open and mechanistic studies are still missing.

In the frame of bioprospecting initiatives, investigations on the diversity of macrofungi in Greece have been intensified over the last 20 years, and to date, more than 2800 species have been recorded [30], including edible cultivated species which are studied both for their use in biotechnological applications and for production of mushrooms with improved organoleptic and nutritional characteristics [31,32,33]. Since basidiomycetes, in particular, contain high β-glucan levels, ongoing studies aim at screening autochthonous fungal resources and selecting species/strains with the potential to yield novel products and/or develop new applications, with emphasis on nutraceuticals.

In this work, we investigated the genoprotective effects of *Pleurotus eryngii, Pleurotus ostreatus* and *Cyclocybe cylindracea*, after having screened several strains of basidiomycetes in respect to their β-glucan content and mushroom cultivation performance, following their in vitro fermentation by faecal inocula from healthy volunteers. In parallel, we assessed the global metabolic profiling of the pre- and post-fermentation supernatants (FSs) by the use of proton nuclear magnetic resonance (^1^H-NMR) spectroscopy.

## 2. Results

### 2.1. Evaluation of Fungal Strains

**Figure 1:**
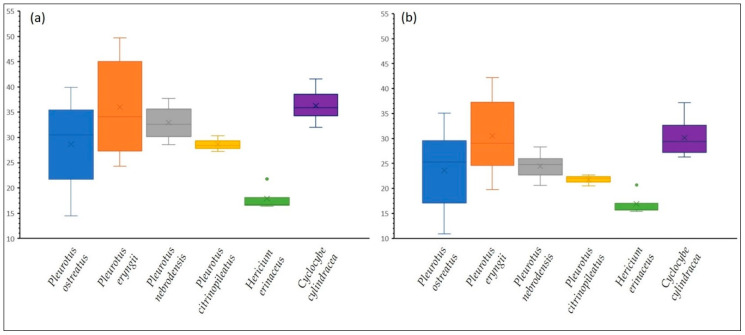
Box plots depicting (**a**) total and (**b**) β-glucan content (%, dry weight [d.w.]) of mushroom species cultivated in various substrates. The horizontal line in each box represents the median, the x represents the mean, the rectangle represents the second and third quartile. An outlier (green dot) is shown among *Hericium erinaceus* cases.

In the frame of a preliminary screening assessment, 29 strains of *Pleurotus ostreatus*, *P. eryngii*, *P. nebrodensis*, *P. citrinopileatus*, *Hericium erinaceus* and *Cyclocybe cylindracea* were cultivated on various substrates (i.e., wheat straw (WS) or beech sawdust (BS), and in their mixtures in various ratios (*w*/*w* fresh weight [f.w]) with grape marc (GM) or olive prunings (OLPR), and of olive leaves (OL) with two-phase olive-mill waste (TPOMW)) and comparatively evaluated with respect to their biological efficiency (i.e., ratio of mushrooms’ fresh weight to the respective substrates’ dry weight), productivity (i.e., ratio of biological efficiency over the time length of the cultivation period) and mushrooms’ glucans content. The results are presented in Supplementary Table S1. Summarized results per species studied regarding total glucan and β-glucan content are depicted in Figure 1. The highest content of total and β-glucans (i.e., 49.7% and 42.2%, respectively) was observed in *P. eryngii* mushrooms (strain LGAM 216) cultivated on substrates consisting of WS:GM (1:1). *Cyclocybe cylindracea* (e.g., CC2, CC493 and CC505) cultivated in WS presented the second highest content in glucans, followed by *P. ostreatus* strains 1123 and LGM 22 cultivated in substrates containing OLPR:TPOMW (1:1). It is noteworthy that several strains led to the production of mushrooms with increased β-glucan content when cultivated on olive by-products (i.e., OL, OLPR and/or TPOMW) as compared to WS only, which is the commonly used substrate. Such enhancement in glucan content of mushrooms produced on by-products of olive industries or wineries was previously reported for *Pleurotus* spp. [13] and *Hericium erinaceus* [34].

Apart from total and β-glucan contents, biological efficiency and productivity were also taken into account for the selection of fungal strains for further comparative analysis, since these cultivation parameters are indicative of the investigated strains’ potential for bioprospecting and commercial exploitation. Hence, five fungal strains cultivated on WS substrate were selected to be fermented in vitro, i.e., *P. ostreatus* strains 1123 and LGM 22, *P. eryngii* strain LGAM 216 and *C. cylindracea* strains CC2 and CC505.

### 2.2. Inhibition of Cell Proliferation and Genoprotection

#### 2.2.1. Pilot Study with P. ostreatus Strain 1123

**Figure 2:**
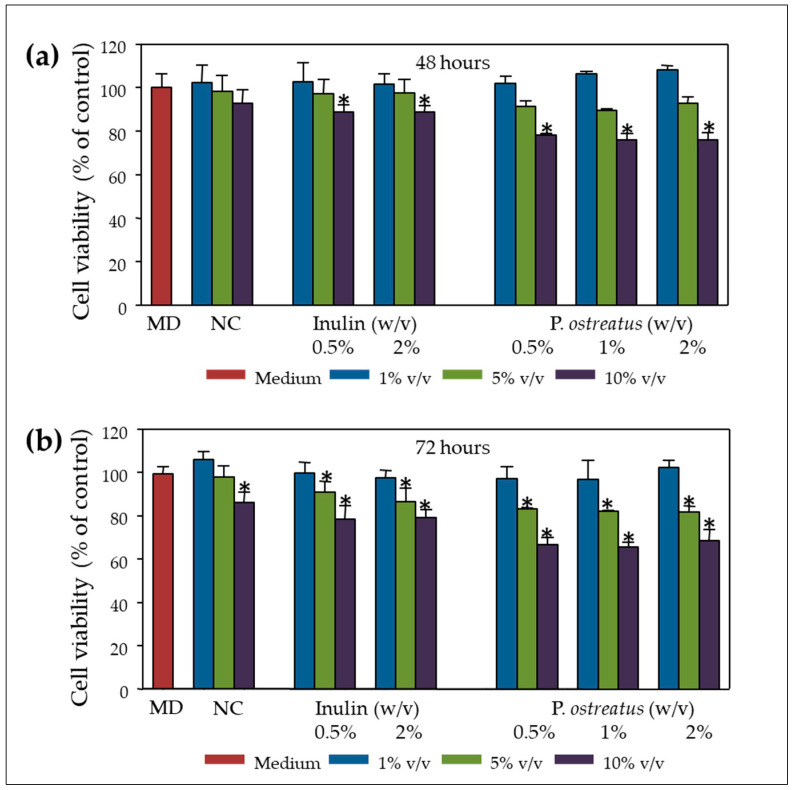
Inhibition of cell proliferation by post-fermentation supernatants (FSs): FSs with different concentrations of the prebiotic inulin (0.5% *w/v* and 2% *w/v*) and lyophilized P. ostreatus 1123 powder (0.5% *w/v*, 1% *w/v* and 2% *w/v*), fermented by fecal inocula from one healthy donor were incubated with Caco-2 cells at 1%, 5% and 10% *v/v* for 48 (**a**) and 72 h (**b**)**.** Cells treated with medium only (MD) served as control. NC: basal medium with no additional carbohydrate source. All values are expressed as the mean ± standard deviation (SD) of at least two independent experiments. * *p* < 0.05 versus control/non-treated cells. Associations were not significant unless otherwise indicated.

In the context of a pilot study, in vitro static batch fermentations were carried out for 24 h, as previously described [35], in order to evaluate the effect of fermentation supernatants (FSs) on Caco-2 cell proliferation and subsequently study their genotoxicity. Batch fermentations were performed with different concentrations of the prebiotic inulin (INU) (0.5% *w/v* and 2% *w/v*) and lyophilised *P. ostreatus* 1123 powder (0.5% *w/v*, 1% *w/v* and 2% *w/v*), utilising faecal inocula from one healthy donor. FSs were obtained and incubated with human colon adenocarcinoma Caco-2 cells at 1%, 5% and 10% *v/v* of culture medium for 48 and 72 h. As shown in Figure 2a,b, FS of inulin and *P. ostreatus* exhibit cytotoxic effects mainly at higher concentrations (10% *v/v*) and longer incubation time (72 h). The concentration of inulin and *P. ostreatus* powder in the in vitro static batch culture did not affect the cytotoxic potential of the fermentation supernatants. FS cytotoxicity was further verified in human monocytic U-937 cells, which proved even more sensitive than Caco-2 cells (data not shown).

**Figure 3:**
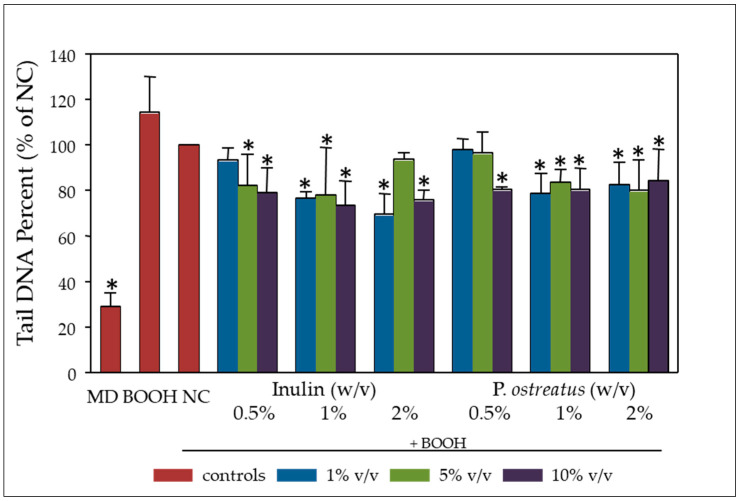
Genoprotective effect of fermentation supernatants (FSs): Fermentations were performed at increasing concentrations 0.5%, 1% and 2% *w/v* inulin or lyophilized *P. ostreatus* 1123 and with no additional carbohydrate source (NC) as a control. Caco-2 cells were incubated with NC, inulin (INU) and *P. ostreatus* (PO) FS at 1%, 5% and 10% *v/v* of culture medium for 48 h. The genotoxic agent tert-butyl hydroperoxide (BOOH) (500 μΜ) was added one hour prior to harvesting. Cells in medium (MD) and cells treated only with BOOH were used as negative and positive controls, respectively. All values are expressed as the mean ± SD of two independent experiments. * *p* < 0.05 versus NC. NC: basal medium with no additional carbohydrate source. Associations were not significant unless otherwise indicated.

The effect of increasing concentrations of fermentation supernatants on the DNA damage induced by tert-butyl hydroperoxide (BOOH), an organic peroxide widely used to induce oxidative DNA damage [36], after 48 h of pre-incubation in Caco-2 cells, is presented in Figure 3. Both inulin and *P. ostreatus* revealed significant genoprotective activities at all tested concentrations (1%, 5% and 10% *v/v*) compared to the controls NC (no additional carbohydrate source added) and BOOH (no fermentation supernatant added). It is noteworthy that even at the non-cytotoxic concentration of 1% *v/v*, the genoprotective effect of the post-fermentation supernatants was evident.

#### 2.2.2. Validation Study

**Figure 4:**
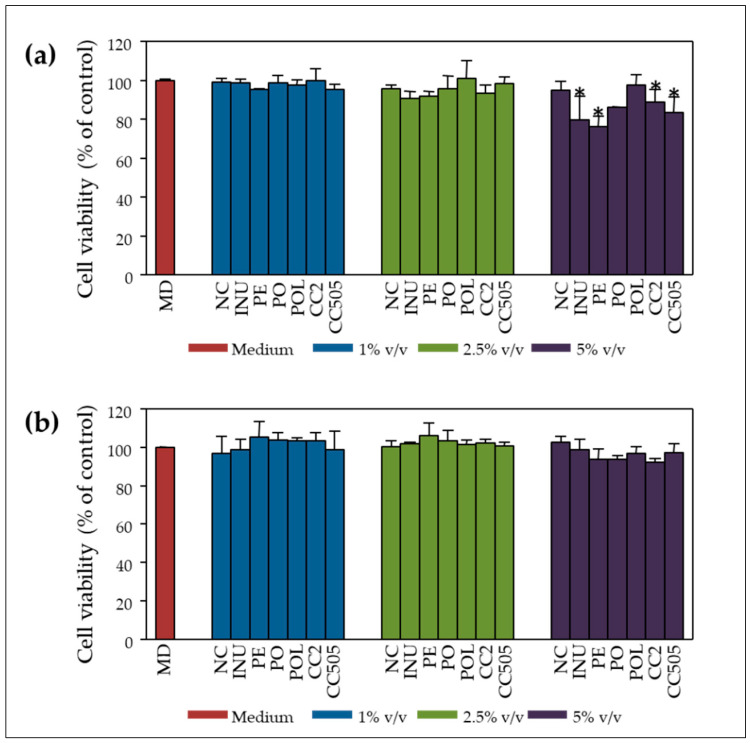
Inhibition of cell proliferation by pre- and post-fermentation supernatants (FSs): Caco-2 cells were treated with 1%, 2.5% and 5% *v/v* of (**a**) pre-fermentation (t = 0 h) and (**b**) post-fermentation (t = 24 h) supernatants of selected mushrooms for 48 h. Cells treated with medium only served as control (MD). NC: basal medium with no additional carbohydrate source, INU: inulin, PO: *P. ostreatus* strain 1123, POL: *P. ostreatus* strain LGM 22, PE: *P. eryngii* strain LGAM 216, CC2: *C. cylindracea* strain CC2, CC505: *C. cylindracea* strain 505. Faecal inocula derived from 8 donors and the fermentation supernatants were pooled for this process. All values are expressed as the mean ± SD of at least two independent experiments. * *p* < 0.05 versus control/non-treated cells. Associations were not significant unless otherwise indicated.

Based on the above results, we proceeded to in vitro static batch fermentations for 24 h, as previously described [36], in the presence of lyophilised powder of the selected mushrooms (*P. ostreatus* strains 1123 and LGM 22, *P. eryngii* strain LGAM 216 and *C. cylindracea* strains CC2 and CC505) at a concentration of 2% (*w/v*) of the basal medium and faecal inocula from eight (8) asymptomatic donors (>65 years). After 24 h fermentation, total bacterial levels significantly increased in nearly all substrates compared to NC. Inulin and all tested mushrooms demonstrated positive mean Prebiotic Indexes (PIs), with higher levels detected in the case of the prebiotic inulin, *P. eryngii* and *C. cylindracea* (CC2, CC505) mushrooms. Inulin (*p* = 0.005) and *P. eryngii* (*p* = 0.021) resulted in significantly higher PIs compared to NC and induced positive PIs results in all 8 runs of the experiment [35]. The effect of pooled pre- (t = 0 h) or post-fermentation (t = 24 h) supernatants on Caco-2 cells’ viability was evaluated at increasing concentrations of 1%, 2.5% and 5% *v/v* after 48 h of incubation. As shown in Figure 4a, inulin, *P. ostreatus*, *P. eryngii* and *C. cylindracea* (CC2 and CC505) pre-fermentation supernatants (t = 0 h) significantly decreased cell viability levels at a concentration of 5% *v/v* compared to non-treated cells and NC, whereas concentrations <5% *v/v* did not affect the rate of cell proliferation. Interestingly, post-fermentation supernatants of all mushrooms tested at the same concentration (5% *v/v*) restored the rates of cell proliferation (Figure 4b).

**Figure 5:**
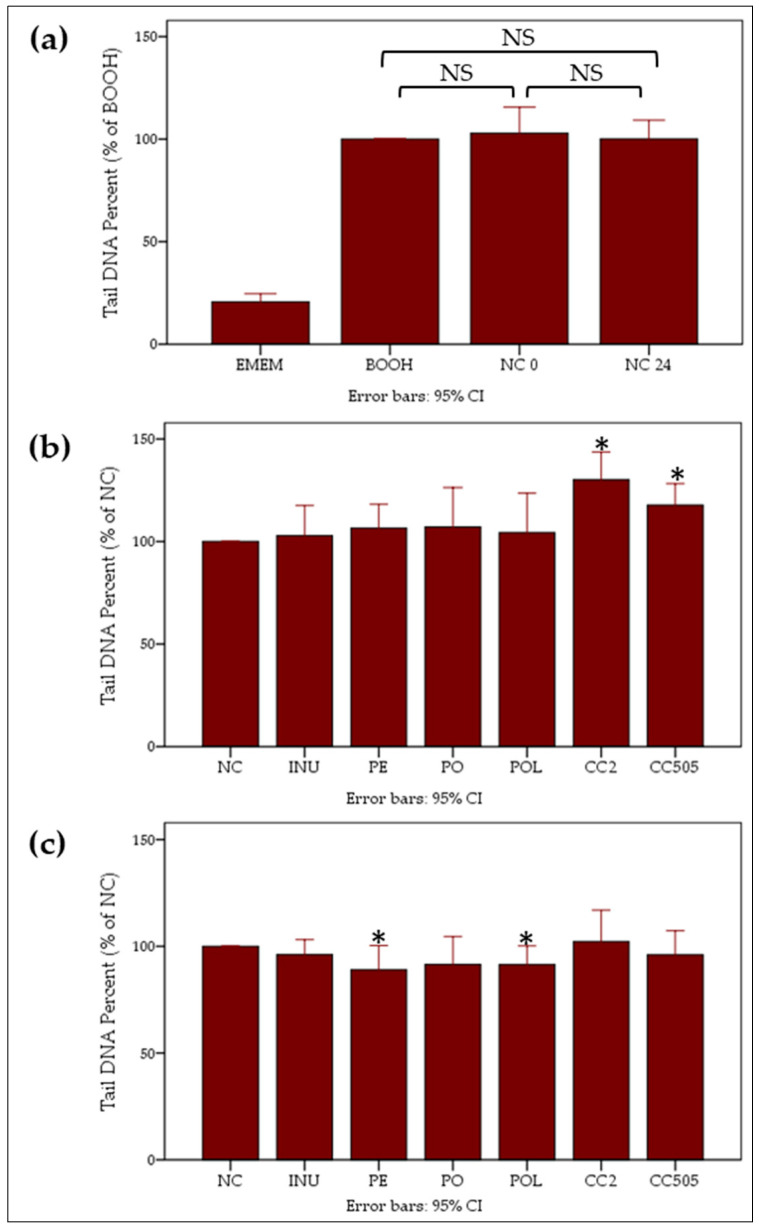
Genotoxic and genoprotective effects of pre- and post-fermentation supernatants. Caco-2 cells were treated with 1% *v/v* pre- and post-fermentation supernatants of NC, INU, PO, POL, PE, CC2 and CC505 for 24 h. The genotoxic agent BOOH (500 μM) was added for one hour. (**a**) genotoxic effect of BOOH, NC 0 and NC 24 (% of BOOH), (**b**) genotoxic effect of pre-fermentation supernatants (% NC t = 0 h), (**c**) genoprotective effect of post-fermentation supernatants (% NC t = 24 h). NC: basal medium with no additional carbohydrate source, INU: inulin, PO: *P. ostreatus* strain 1123, POL: *P. ostreatus* strain LGM 22, PE: *P. eryngii* strain LGAM 216, CC2: *C. cylindracea* strain CC2, CC505: *C. cylindracea* strain CC505. All values are expressed as the mean ± 95% CI (Confidence Interval). * *p* < 0.05 versus control (paired *t*-test). Associations were not significant unless otherwise indicated.

Thereafter, Caco-2 cells were incubated with the pre- and post-fermentation supernatants from the 8 donors separately, for 24 h, and subsequently challenged with the genotoxic agent BOOH for one hour. According to our results, there was no effect of the NC pre- and post-fermentation supernatants on BOOH-induced DNA damage levels (Figure 5a). Moreover, the pre-fermentation supernatants of CC2 and CC505 exhibited significantly increased genotoxicity compared to NC (Figure 5b). These data indicate that the water-soluble constituents of *C. cylindracea* exhibit genotoxic activities which are inactivated or counteracted after the fermentation process. The post-fermentation supernatants of *P. eryngii* and *P. ostreatus* LGM 22 exhibited a significant genoprotective activity compared to NC (Figure 5c; Table 1). A similar trend was observed for the FS of *P. ostreatus* strain 1123 and inulin but was not significant. Overall, it seems that after 24 h of in vitro fermentation, genotoxic mushrooms’ constituents, like those observed in the case of *C. cylindracea,* are either degraded or counteracted by genoprotective compounds produced by gut microbiota (Figure 5c).

**Figure 6:**
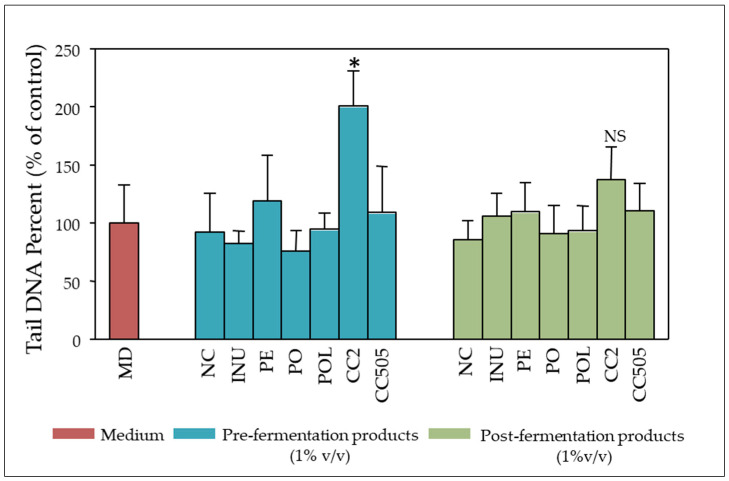
Direct genotoxic effects of pre- and post-fermentation samples: Caco-2 cells were treated with 1% *v/v* pre- and post-fermentation samples of NC, INU, PO, POL, PE, CC2 and CC505. Cells treated with culture medium only served as control (MD). NC: basal medium with no additional carbohydrate source, INU: inulin, PO: *P. ostreatus* strain 1123, POL: *P. ostreatus* strain LGM 22, PE: *P. eryngii* strain LGAM 216, CC2: *C. cylindracea* strain CC2, CC505: *C. cylindracea* strain CC505. All values are expressed as the mean ± SD of two independent experiments. * *p* < 0.05 versus medium. Pooled samples from 8 donors were used. Associations were not significant unless otherwise indicated.

In order to confirm the genotoxic potential of *C. cylindracea* strains CC2 and CC505, Caco-2 cells were incubated with pre- and post-fermentation supernatants (PO, POL, PE, CC2, CC505, INU, NC of all 8 donors) at non cytotoxic concentrations (1% *v/v* culture medium), for 24 h without any BOOH challenge. Subsequent comet assay analysis confirmed that the pre-fermentation supernatant of *C. cylindracea* strain CC2 (t = 0 h) is significantly genotoxic, while the corresponding post-fermentation supernatant is not (Figure 6), which suggests that genotoxic constituents are indeed either degraded or counteracted in the process of the in vitro fermentation.

### 2.3. NMR-Based Metabolomics Profiling

**Figure 7:**
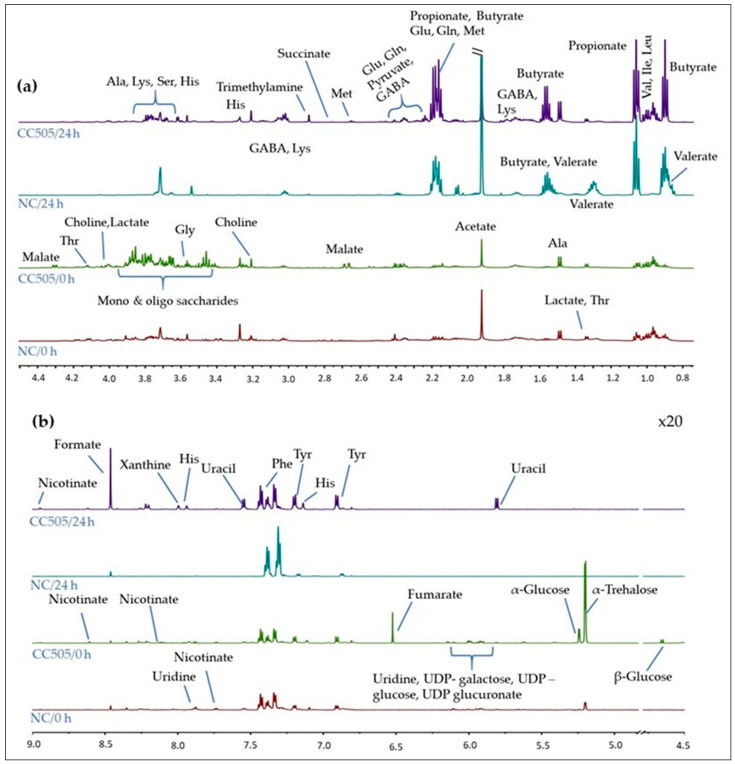
Representative ^1^H-NMR spectra of the pre- (t = 0 h) and post-fermentation (t = 24 h) supernatants from one volunteer, for both NC (basal medium with no additional carbohydrate source) and *C. cylindracea* CC505 (**a**) 0.8–4.4 ppm, and (**b**) 4.5–9.0 ppm. NC: basal medium with no additional carbohydrate source, CC505: *C. cylindracea* strain 505.

A preliminary untargeted NMR-based metabolomics study of *P. ostreatus*, *P. eryngii* and *C. cylindracea* fermentation supernatants (FSs) was conducted including the pre- and post-FSs from only 3 randomly selected volunteers out of the total 8. Thirty-seven (37) metabolites were unambiguously assigned with the use of two-dimensional (2D) homo- and hetero-nuclear NMR experiments, sophisticated tools such as Metabominer and Chenomx Profiler and literature data, as described in the Section 4. Among them, amino acids, organic acids, including short chain fatty acids (SCFAs), mono- and oligo-saccharides and nucleotide sugars, were identified. ^1^H-NMR spectra of pre- and post-FS pairs of NC and *C. cylidraceae* 505 with indications to the identified metabolites are presented in Figure 7.

**Figure 8:**
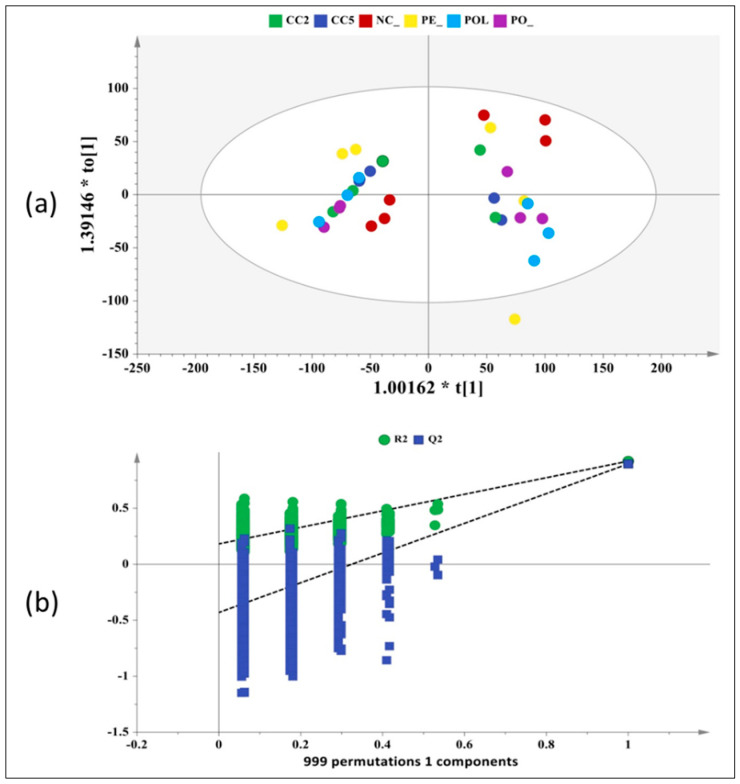
Illustration of multivariate statistical analysis of the ^1^H-NMR data for the pre- and post-fermentation samples from 3 randomly selected volunteers. (**a**) Orthogonal projections to latent structures with discriminant analysis (OPLS-DA) scores map. (R2X(cum) = 0.68, (R2X(cum) = 0.92, Q2(cum) = 0.90, Pareto scaling, Hotelling T2 = 95%). (**b**) Validation of the OPLS-DA model by permutation analysis indicating that the extracted model is significantly different from a model built on random data. The permutation tests were carried out with 999 random permutations, thus providing significance of the model at the 0.05 level. PO: *P. ostreatus* strain 1123, POL: *P. ostreatus* strain LGM 22, PE: *P. eryngii* strain LGAM 216, CC2: *C. cylindracea* strain CC2, CC505: *C. cylindracea* strain CC505.

**Figure 9:**
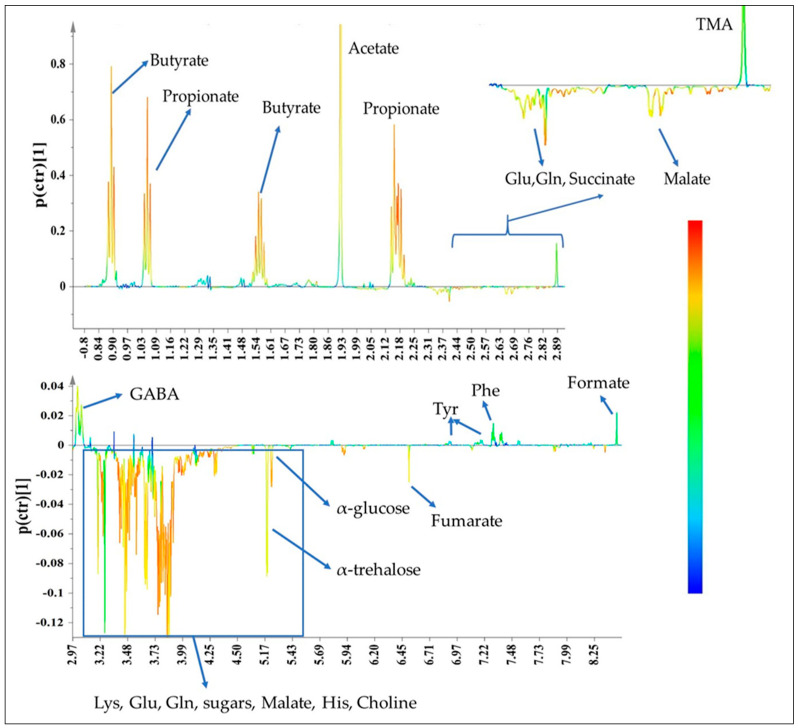
S-line plot based on the OPLS-DA model in order to visualise the most discriminative metabolites. The colour coding corresponds to the significance of their contribution with the red colour depicting the metabolites that most influence the separation of the groups. Resonance lines with positive values correspond to the most characteristic metabolites for the post-fermentation supernatants, and those with negative values constitute the most discriminative for the pre-fermented supernatants. PO: *P. ostreatus* strain 1123, POL: *P. ostreatus* strain LGM 22, PE: *P. eryngii* strain LGAM 216, CC2: *C. cylindracea* strain CC2, CC505: *C. cylindracea* strain CC505.

Unsupervised Principal Component Analysis (PCA) illustrated a clear separation and segregation of the pre- and post-fermentation supernatants across the PC1 (R^2^X(cum) = 0.70, Q^2^(cum) = 0.60), mirroring the metabolic variance arising from the fermentation process (Supplementary Figure S1). This allowed the application of supervised orthogonal projections to latent structures with discriminant analysis (OPLS-DA) using the time point as the response variable and the matrix of ^1^H-NMR experimental data as independent variables (Figure 8). The corresponding S-line plot enabled the visualization of the metabolites that contributed most to the segregation and discrimination of the two groups through colour coding (Figure 9).

As evidenced by the ^1^H-NMR data and the multivariate analysis (S-line plot), a number of metabolic alterations occurred in post-fermentation supernatants as a result of faecal microbiota metabolism. In vitro fermentation resulted in a remarkable increase of short chain fatty acids (SCFAs), namely acetate, propionate, butyrate and formate, and a concomitant decrease of the mono- and oligo-saccharides content. Furthermore, malate and fumarate were detected in the pre-fermentation supernatants only when mushrooms were present. As mushroom constituents, they were apparently exhaustively consumed in the course of the fermentation process. Interestingly, the in vitro fermentation process was also associated with significant alteration in the concentration of the aromatic amino acids phenylalanine (Phe) and tyrosine (Tyr) as well as of the nucleobase uracil. It is also worth noting that the use of mushrooms as an additional carbon source enhanced the production of trimethylamine (TMA) at the expense of choline as well as of the neurotransmitter gamma-aminobutyric acid (GABA).

## 3. Discussion

Mushroom species have been known for their health-promoting properties for centuries now. On the other hand, in recent years, the crucial role of fungal polysaccharides in enhancing the immune system, preventing cancer, hyperglycaemia and hypercholesterolemia is being documented [2,3,4,5]. In edible mushrooms, β- (1 → 3) -D-glucans and especially β- (1 → 3, 1 → 6)-d-glucans, a large group of biopolymers, essential components of the cell wall, are considered candidate prebiotics since the populations of beneficial bacteria increase as a result of their interaction with gut microbiome [15]. Nevertheless, the mechanisms underlying the link between the anticancer properties of edible mushrooms and possible prebiotic activity have not been elucidated yet [16]. A main issue, still open, is to what extent metabolites produced by the gut microbiota during the digestion of edible mushrooms are responsible for their beneficial effects. Very few studies exist so far on gut microbiota-mediated fermentation of dietary fibres and its role in the protection of colonic cells from genotoxic insults [24,25,26,27,28]. Although the results overall are not conclusive, inulin fermentation supernatants seem to confer resistance to genotoxic agents by elevating phase II detoxification enzymes [28,37], and inulin-type fructans’ consumption reduces colorectal cancer risk [38,39].

The aim of this study was to investigate the anti-genotoxic properties of the in vitro fermentation supernatants of edible cultivated mushroom species, with emphasis on material collected from Greek habitats characterised by high β-glucan content. The effect of mushroom fermentation by gut microbiota is depicted in the metabolites produced during this process. Therefore, the global metabolomic profile of their fermentation supernatants was assessed by ^1^H-NMR spectroscopy in an effort to identify biomarkers potentially associated with health-promoting effects of edible mushrooms, emphasising on potential genoprotective effects. To our knowledge, this is the first time that the genoprotective action of in vitro fermented edible mushrooms by faecal microbiota has been investigated. There is substantial experimental evidence supporting the genoprotective effect of mushrooms and β-glucans in vitro [17,18,19], in vivo [20,21,22] and in polypectomised patients [23] but there is no evidence of experimentation falling within the concept of fermentation by intestinal microbiota and subsequent production of genoprotective metabolites.

The genera *Pleurotus* and *Cyclocybe*, selected for analysis in the present work, produce mushrooms rich in β-glucans which were very recently shown to exhibit a beneficial influence on the composition of gut microbiota of apparently healthy subjects over 65 years old (i.e., increase of *Bifidobacterium* spp. and *F. prausnitzii* populations) [35]. Interestingly, correlation analysis revealed significant positive associations of mean Prebiotic Indexes values of these mushrooms with their average total glucan and β-glucan content [35]. All fungal strains selected to be used as carbon sources in the respective in vitro static batch culture fermentation were cultivated on a wheat straw-based substrate and produced mushrooms with β-glucan contents exceeding 30% (d.w.) (Figure 1, Supplementary Table S1). The methodological approach applied in the present study “mimics” the fermentation of these fibres in vivo by the colon microbiota and constitutes an effort to bridge the gap between laboratory findings and clinical interventions.

The aqueous phase of human stool (faecal water) is known to be cytotoxic mainly due to the contained bile acids [40]. Furthermore, in vitro fermentation of the mushrooms selected to be analysed in this work, as well as of inulin, resulted in significantly higher levels of total volatile fatty acids (VFAs) [35]. Therefore, it is not surprising that NC and both inulin or *P. ostreatus* 1123 fermentation supernatant (FS) were also cytotoxic to CaCo-2 cells, in a dose- and time-dependent manner (Figure 2). It is worth mentioning that FS cytotoxicity was further verified in human monocytic U-937 cells, which proved even more sensitive than Caco-2 cells. Significant inhibition of cell proliferation was observed at lower doses and shorter incubation periods (data not shown). Furthermore, we investigated possible anti-genotoxic effects of inulin and *P. ostreatus* 1123 FS against the DNA damage induced by BOOH, a model genotoxic agent mainly inducing free radical intermediates of oxidative stress [36,41]. Pre-treatment of Caco-2 cells with FS of both inulin and *P. ostreatus* 1123 for 48 h resulted in a significant decrease in DNA damage induced by subsequent BOOH challenge, for both the non-cytotoxic (1% and 5% FS in cell culture medium) and cytotoxic (10% FS) dosing regimens (Figure 3). Again, similar, even more prominent results were obtained in U-937 cells (data not shown). The observed genoprotection could be a reflection of enhanced cellular metabolism, including stimulation of DNA repair and defence systems against oxidative stress [28,42].

In order to validate the results of the pilot study regarding cytotoxicity/anti-genotoxicity of *P. ostreatus* 1123 in vitro FS, we proceeded with an extended investigation including *Pleurotus* and *Cyclocybe* strains already evaluated as to their β-glucan content and prebiotic potential [35]. The pre-fermentation supernatant (pFS) and FS of the negative control (NC: sample without additional carbon source) were not cytotoxic over the whole range of concentrations used (1%, 2.5%, 5% *v/v*) in the course of cell proliferation (Figure 4). Interestingly, the pFS of mainly *P.ostreatus* 1123 and *P. eryngii* LGAM216 were cytotoxic at the higher concentration of 5% *v/v* of culture medium, while the post-fermentation supernatants respectively, were not cytotoxic (Figure 4). Possibly, the above-mentioned mushrooms contain cytotoxic water-soluble constituents which are either degraded over the 24 h fermentation period or metabolized to non-cytotoxic compounds by the gut microbiota.

The concentrations applied in the comet assay should range from non-cytotoxic to those resulting in approximately 80% viability since DNA breaks can be a secondary effect of cytotoxicity and thus, could produce false-positive results [43]. Therefore, for the validation study, concentrations up to 5 % *v/v* FS/culture medium could have been applied. Nevertheless, and contrary to the outcome of previous studies [24,25,26,27,28], the lowest concentration of 1% *v/v* was adopted in the BOOH-challenged genotoxicity experiments since it is definitely non-cytotoxic and probably more relevant to the in vivo human gut epithelium–gut microbiome interaction [43]. *P. ostreatus* strain 1123 and *P. eryngii* pre-fermentation supernatants did not show any genotoxic activity, whereas the respective *C. cylindracea* CC2 and CC505 supernatants were significantly genotoxic relative to the control NC supernatant (Figure 5b). *C. cylindracea* may contain genotoxic water-soluble ingredients which are either degraded or metabolised to non-genotoxic compounds by the gut microbiota in the process of the 24 h in vitro fermentation. This was confirmed at least for the *C. cylindracea* CC2 when comet assay was performed without BOOH challenge (Figure 6). Even at this very low concentration of fermentation supernatants in the cell culture medium (1% *v/v*) utilised, it was evident that in vitro fermentation of *P. ostreatus* and *P. eryngii* conferred genoprotection to Caco-2 cells against the BOOH-induced DNA strand breaks (Figure 5c). It should be noted that the importance of inter-individual variations in the sensitivity and/or protection against genotoxic agents is clearly indicated in the case of inulin and *P. ostreatus* strain 1123. When faecal inocula from one volunteer were used in the pilot study, FSs of inulin and *P. ostreatus* strain 1123 were genoprotective (Figure 3). In contrast, no significant effect was observed overall in the validation study, where FS represents the mean value from all 8 volunteers (Figure 5).

To the best of our knowledge, this is the first time that the genoprotective action of edible mushrooms has been investigated. The fact that even a very low concentration of fermentation supernatants has a clear protective effect is considered extremely important. The health benefits of high-β-glucan mushrooms are clearly expanding towards the protection of genome integrity, which is fundamental, especially for the elderly who were the focal point of the present study.

As mentioned above, an important issue is the investigation of the role of the gut microbiota metabolome, produced during the digestion of mushrooms’ polysaccharides, in health and disease. Therefore, in an effort to identify metabolites/biomarkers associated with the health-promoting properties of edible mushrooms, an untargeted NMR-based study addressed the global metabolomic profile of the in vitro pre- and post-fermentation samples of the fungal substrates by faecal inocula by the use of ^1^H-NMR spectroscopy. Multivariate data analysis monitored the metabolic variability of the faecal microbiota and probed to the most discriminant biomarkers.

Several fermentation-specific metabolites were identified in the NMR spectra. A remarkable increase in the concentration of the short chain fatty acids (SCFAs) acetate, propionate and butyrate was observed in all post-fermentation supernatants as expected considering the high β-glucan content of *P. ostreatus, P. eryngii* and *C. cylindracea* [35,44,45]. Among them, butyrate is the most important SCFA for the health of the human intestinal epithelium since it is the main source of energy for epithelial cells. There is also evidence that both butyrate and propionate may activate intestinal gluconeogenesis with beneficial effects on glucose and energy homeostasis [46] and has the ability to regulate gene expression by inhibiting histone deacetylases [47]. Acetate, the most abundantly detected SCFA, is also contributing in butyrate biosynthesis. In humans, acetate is transported to peripheral tissues and used in the metabolism and lipogenesis of cholesterol [48]. The observed increase of formate concentration after 24 h fermentation is probably attributed to the high β-glucan content of *P. ostreatus, P. eryngii* and *C. cylindracea* mushrooms [49]. Malate and fumarate are normally detected at trace quantities in faeces due to their extensive utilisation by other bacteria in the pertinent cross-feeding processes [44]. They were detectable in our pre-fermented supernatants, probably because they are water-soluble constituents of mushrooms; however, the fermentation process resulted in their elimination, most likely due to their reduction to propionate.

A significant alteration of the aromatic amino acids phenylalanine (Phe) and tyrosine (Tyr), as well as of the nucleobase uracil, was also observed. These metabolites derive from proteolysis and saccharolysis respectively, and their role regarding the gut microbiota functionality is under investigation. The production of trimethylamine (TMA) was also enhanced in mushrooms’ post-fermentation supernatants in relation to the respective pre-fermentation ones. Mushrooms constitute a source of choline and choline-derivatives [50], as confirmed by the presence of choline in pre-fermentation supernatants. TMA is the product of anaerobic gut microbiota metabolism of dietary choline and its derivatives (betaine) and l-carnitine [51]. TMA is further absorbed through intestinal epithelium and is oxidised in the liver by flavin-containing monooxygenase (FMO) enzyme family-forming thrimethylamine N-oxide (TMAO), which is excreted in the urine. TMAO has been implicated in atherosclerosis and cardiovascular disease [52].

The in vitro fermentation process was also associated with an increase in the production of gamma-aminobutyric acid (GABA). GABA is produced by the decarboxylation of glutamic acid (Glu), which is formed from glutamine (Gln) [53], and both compounds were shown to be reduced in post-fermentation samples. GABA functions as a neurotransmitter in the central nervous system (CNS) by inhibiting the GABA receptors and reducing the activity of neurons. Recent in vitro and in vivo data demonstrated that the abundance of GABA-producing bacteria is correlated with brain neuronal activity influencing the host [54] and disorder in GABA signalling is implicated in a multitude of neurologic and psychiatric conditions [55]. 

The potential role of butyrate and acetate in genoprotection has been previously reported [25]. The synergistic beneficial effect of butyrate with other fermentation metabolites towards the reinforcement of the intestinal barrier integrity has also been suggested [56]. Therefore, towards this end, subsequent thorough ^1^H-NMR profiling will be undertaken in order to distinguish the different fermentation metabolic fingerprints of the diverse mushrooms applied, assess the effect of cultivation substrates used and finally, establish metabolic biomarkers of the fermentation process relevant to human health.

## 4. Materials and Methods

### 4.1. Fungal Strains, Mushroom Cultivation and Determination of Glucans Content

The biological material evaluated for the purposes of this study consisted of 29 strains assigned to six species of Basidiomycota, i.e., *Pleurotus ostreatus*, *P. eryngii*, *P. nebrodensis*, *P. citrinopileatus*, *Hericium erinaceus* and *Cyclocybe cylindracea*. All strains, with the exception of *P. ostreatus* strain CS, *P. citrinopileatus* CS and *P. nebrodensis* UPA 6, were isolated in Greece in the frame of an ongoing investigation on the mushroom diversity. Pure cultures are maintained in the fungal Culture Collection of the Laboratory of General and Agricultural Microbiology (Agricultural University of Athens, Greece).

Among the strains examined, 14 were assessed for the first time with respect to mushroom production performance and glucans content, while the rest were evaluated in the frame of previous pertinent studies [13,30,31,32]. Mushroom cultivation was conducted in substrates consisting of wheat straw (WS) or beech sawdust (BS), and in their mixtures in various ratios (*w/w* f.w.) with grape marc (GM) or olive prunings (OLPR), and of olive leaves (OL) with two-phase olive-mill waste (TPOMW) (Supplementary Table S1). Prior to cultivation, cereal grain spawn was prepared as previously described [57]. Polypropylene autoclavable bags were then filled with 1 kg of the substrate (moisture content ranging from 50% to 68%) and sterilised at 121 °C (1.1 atmospheres) for 1 h. Substrates were then inoculated with spawn at a rate of 5% w/w, in four replicates per substrate/strain combination. Incubation of cultures and mushroom production was performed in special cultivation chambers as previously described [32]. Six to ten weeks were required to complete the cultivation cycle (including two production flushes) depending on the combination of substrate and fungal strain, while at the end of the cultivation period, the mushroom yield was measured and expressed as biological efficiency (defined as the ratio of mushrooms’ fresh weight to the substrates’ dry weight). In addition, productivity, expressed as the ratio of the biological efficiency value obtained over the time length (in days) of the respective cultivation period, was also calculated.

Harvested mushrooms were freeze-dried, ground to a particle size less than 2 mm and stored at −20 °C prior to analyses. The determination of mushrooms’ content in total and α-glucans was conducted using the Mushroom and Yeast Beta-Glucan assay kit (Megazyme Int., Bray, Ireland), while the β-glucan content was calculated as the difference between the total and α-glucans.

### 4.2. Faecal Sample Collection and In Vitro Static Batch Culture Fermentations

Faecal donors were apparently healthy subjects (>65 years), meeting appropriate inclusion criteria, as previously described [35]. The study was conducted according to the guidelines laid down in the Declaration of Helsinki and under the approval of the Bioethics Committee of Harokopio University, Athens, Greece (62-03/07/2018). Written informed consent was obtained from all faecal donors prior to their inclusion in the study. Faecal sample collection, preparation of faecal inocula and the in vitro static batch culture fermentation process were performed as previously described [35].

### 4.3. Cell Culture and Treatment

Human small intestinal cells Caco-2 (Caco2) (ATCC^®^-HTB-37^TM^) and human monocytic U-937 cells (ATCC^®^ CRL-1593.2™) were grown in EMEM and RPMI media respectively (containing phenol red, nonessential amino acids, 1 mM sodium pyruvate, 2 mM l-glutamine, 1 g/L glucose and 1.5 g/L sodium bicarbonate), supplemented with 20% fetal bovide serum (FBS) and 1% penicillin/streptomycin (Gibco-Life Technologies, Waltham, MA, USA), at 37 °C in a humidified incubator with 5% CO_2_. The cells were maintained as a monolayer culture for 24 h before each treatment.

### 4.4. Cell Proliferation Sssays

Cell viability was assessed using two analogous assays, the 3-(4,5-Dimethyl-2-thiazolyl)-2,5-diphenyl-2H-tetrazolium-bromide (MTT), purchased from Serva (Heidelberg, Germany), and the PanReac AppliChem Cell Proliferation Kit XTT (sodium 3’-[1-(phenylaminocarbonyl)-3,4-tetrazolium]-bis(4-methoxy6-nitro) benzene sulfonic acid hydrate), according to the manufacturers’ standard protocols. Caco-2 cells were plated in 96-well plates (1.3 × 10^4^ cells/well), treated with different concentrations of faecal slurries and incubated for different time intervals. Cell viability was measured at 470 nm (690 nm for background) using a Safire II, TECAN microplate reader (Grödig, Austria).All assays were carried out in triplicate and non-treated cells were used in all cases as a control.

### 4.5. Comet Assay

Caco-2 cells were seeded in a 24-well plate at a density of 1 × 10^5^ cells per well and treated with different amounts of pre- and post-fermentation faecal slurry samples and different incubation periods. Tert-butyl hydroperoxide (BOOH), purchased from Sigma Aldrich (Taufkirchen, Germany), was added in all samples as a standard genotoxicity inducer and incubation was extended for one more hour. Cells cultured without BOOH served as a negative control.

DNA damage was assessed using the alkaline comet assay as described by Turunen et al. [23] with minor modifications. Ten μL of cell suspension (2 × 10^4^ cells) was mixed with 130 μL of 1% (*w/v*) low-melting agarose (LMA) at 37 °C, layered onto agarose-precoated slides, covered with coverslips and kept at 4 °C to solidify. Cell lysis was subsequently performed with ice-cold lysis solution (2.5 M NaCl, 100 mM ethylenediaminetetraacetic acid (EDTA), 10 mM Tris, 1% Triton X-100, pH 10) for 2 h. The electrophoresis was performed at 25 V and 255 mA in a buffer solution containing 1 mM EDTA and 300 mM NaOH (pH > 13) for 30 min at 4 °C. Slides were then washed once with neutralising buffer (0.4 M Tris, pH 7.5) and twice with double distilled sterile water at 4 °C for 10 min each. Slides were immersed in 0.0002% SYBR^TM^ Gold solution (SYBR^TM^ Gold nucleic acid gel stain purchased from Life Technologies Corporation, located in Eugene, OR, USA)in TE buffer (10mM Tris, pH 7.4; 1mM EDTA, pH 8) and examined under a fluorescent microscope (NICON).

Images of the fluorescently stained cell nuclei were analysed using the TriTek CometScore Freeware v 1.5 Imaging software (Waukesha, WI, USA). One hundred nucleoids were analysed per slide. The % DNA present in the tail was chosen as the effect parameter. The Tail DNA is expressed as a percentage of the total DNA content based on the overall fluorescence intensity.

### 4.6. NMR-Based Metabolomics

#### 4.6.1. Sample Preparation

The study group consisted of 34 samples at zero time-point (pre-fermentation; *n* = 17) and 24 h (post-fermentation; *n* = 17). 1.5 mL of fermentation mixture were transferred in 2 mL Eppendorf centrifuge tubes and the samples were stored overnight at −80 °C prior to lyophilisation. Samples were freeze-dried under a vacuum for 24 h at a constant temperature of 25 °C until dryness. The freeze-dried samples were then stored at −80 °C. The samples were defrosted at ambient temperature 30 min prior to NMR experiments and were then dissolved in 540 μL of phosphate buffer (NaH2PO4/Na2HPO4, pH = 7.2) using 60 μL of TSP (0.5 mM) as an internal standard.

#### 4.6.2. NMR Spectroscopy

NMR experiments were performed at 25 °C on a Varian 600 MHz spectrometer (Varian/Agilent Technologies, Palo Alto, California, USA, business closed) using a 1H{13C/15N} 5 mm PFG Automatable Triple Resonance probe. The global metabolic profiling of the studied fermented products was assessed by the application of *1D* nuclear Overhauser enhancement spectroscopy with a mixing time of 200 ms and performing solvent suppression by presaturating the sample with gammaB1 of 103 Hz for 1 s (1D NOESY presat pulse sequence). The NOESY presat experiments were recorded with 64k complex data points and 32 scans. Inverse recovery experiments specified the relaxation delay to 28 s. Total correlation spectroscopy (2D zTOCSY with 256 increments, 64 scans/increment, spectral width 0–11 ppm), and gradient heteronuclear single quantum coherence with adiabatic shaped pulses (2D gHSQCad with 256 increments, 96 scans/increment) experiments were also performed for metabolite identification purposes.

#### 4.6.3. Interpretation of Spectra—Metabolites Assignment

The interpretation of 1D and 2D NMR spectra was performed using Mnova v.14.1 software (Mestrelab Research, S.L., Santiago de Compostela, Spain). 1D NOESY presat spectra were reduced into buckets of 0.001 ppm. The D2O (4.6–4.8 ppm) region was removed. The spectra were normalised to the area of the TSP peak, aligned relative to the TSP peak (0.0 ppm), converted to .csv format and imported to Simca 14 software (UMETRICS, Sartorius Stedim Data Analytics AB, UMEÅ, Sweden) for multivariate statistical analysis (UMETRICS). Metabolites identification was enabled via Metabonimer platform [58] and Chenomx NMR Suite 7.0. (Chenomx Inc., Edmonton, AB, Canada) in combination with literature data [59]. 2D TOCSY and HSQC data were imported in Mnova software and a peak picking list was exported to Metabominer which generated the experimental spectra. The spectra were screened against Metabominers’ biofluid reference library and the derived patterns were examined in order to uniquely identify the corresponding metabolites. A low cut-off threshold was used during the process in order to avoid substantial artifacts as results of baseline distortions, intense solvent lines, ridges, etc. Chenomx Profiler module was used in order to further validate the assigned metabolites.

### 4.7. Statistical Analysis

The data for the prebiotic index, cytotoxic and genotoxic comparisons were analysed using Student’s *t*-test and paired *t*-test using the software SigmaPlot 13.0 (Systat Software, Inc., San Jose, CA, USA) for Windows. The level of significance was set at *p* < 0.05.

For the NMR Multivariate Data Analysis, the matrix of the processed NMR spectra was submitted to the Simca 14 software. Principal Component Analysis (PCA) was applied in order to acquire a comprehensive insight and visualise any relation (trends, outliers) among the observations (samples). The spectral data was scaled using the Pareto algorithm (Par) in order to reduce the influence of intense peaks. The PCA model was extracted at a confidence level of 95%. The dataset was then subjected to OPLS-DA analysis to improve model visualisation and interpretation. The OPLS-DA model was extracted at a confidence level of 95% and Pareto scaled (Par). The S-line plot enabled the detection of the metabolites that influence most group’s identification and segregation. The quality of the models was described by the goodness-of-fit R2 and the predictive ability Q2 values [60].

## 5. Conclusions

In the course of this study, the genoprotective effects of edible mushrooms produced by *Pleurotus eryngii, P. ostreatus* and *Cyclocybe cylindracea* (Basidiomycota) were investigated. Mushrooms from selected strains of these species were fermented in vitro using faecal inocula from eight elderly (>65 years), healthy volunteers. Furthermore, the global metabolic profile of fermentation supernatants was assessed by ^1^H-NMR spectroscopy, in an effort to identify metabolites/biomarkers associated with the health-promoting properties of edible mushrooms.

The main conclusions drawn from the study are the following:

The genoprotective action of edible mushrooms of the genus *Pleurotus* has been documented for the first time, and in vitro fermentation supernatants exhibited a clear protective effect against BOOH-induced DNA damage.

The production of several fermentation-specific metabolites was considerably enhanced. These metabolites included, besides short chain fatty acids (SCFAs) such as acetate, propionate and butyrate, aromatic amino acids (Phe, Tyr), trimethylamine (TMA) and gamma-aminobutyric acid (GABA).

Overall, the above findings provide substantial evidence that edible mushrooms may contain ingredients protecting genome integrity, which is fundamental, especially for healthy ageing. Moreover, the ^1^H-NMR spectroscopy findings pave the way for a subsequent thorough H-NMR profiling to be undertaken in order to establish metabolic biomarkers of the fermentation process relevant to human health.

## Figures and Tables

**Table 1:** Reduction of % DNA in comet tail relative to the control. Paired samples *t*-test.

Paired Differences								
Samples	N	Mean	SD	Interval of the Lower Upper		t	Df	Sig. (2-Tailed)
PE	8	10.80	11.36	1.30	20.30	2.69	7.00	0.03
PO	8	6.88	13.92	−4.76	18.52	1.40	7.00	0.20
POL	8	7.66	9.16	0.01	15.32	2.37	7.00	0.05
CC2	7	−2.23	15.93	−16.96	12.50	−0.37	6.00	0.72
CC505	7	4.00	12.25	−7.33	15.33	0.86	6.00	0.42
INU	8	7.35	12.10	−2.76	17.47	1.72	7.00	0.13

PE: *P.* *eryngii* strain LGAM 216, PO: *P. ostreatus* strain 1123, POL: *P. ostreatus* strain LGM 22, CC2: *C. cylindracea* strain CC2, CC505: *C. cylindracea* strain 505, INU: inulin. Df: degrees of freedom, Sig. (2-Tailed): *p* value from paired *t*-test.

## Supplementary Materials

The following are available online: Figure S1: PCA analysis of the NMR data for the pre- and post-fermentation supernatants, Table S1: Biological efficiency, productivity and total, α- and β-glucans content of *Pleurotus ostreatus*, *P. eryngii*, *P. nebrodensis*, *P. citrinopileatus*, *Hericium erinaceus* and *Cyclocybe cylindracea* strains.
