# Correction: Boulaka et al. Genoprotective Properties and Metabolites of β-Glucan-Rich Edible Mushrooms Following Their In Vitro Fermentation by Human Faecal Microbiota. *Molecules* 2020, *25*, 3554

**DOI:** 10.3390/molecules28145337

**Published:** 2023-07-11

**Authors:** Athina Boulaka, Paris Christodoulou, Marigoula Vlassopoulou, Georgios Koutrotsios, Georgios Bekiaris, Georgios I. Zervakis, Evdokia K. Mitsou, Georgia Saxami, Adamantini Kyriacou, Maria Zervou, Panagiotis Georgiadis, Vasiliki Pletsa

**Affiliations:** 1 Institute of Chemical Biology, National Hellenic Research Foundation, 11635 Athens, Greece; aboulaka@eie.gr (A.B.); pchristodoulou@eie.gr (P.C.); mvlassopoulou@eie.gr (M.V.); mzervou@eie.gr (M.Z.); 2 Department of Biochemistry and Biotechnology, University of Thessaly, 41500 Larissa, Greece; 3 Department of Nutrition and Dietetics, Harokopio University, 17676 Kallithea, Greece; emitsou@hua.gr (E.K.M.); kyriacou@hua.gr (A.K.); 4 Laboratory of General and Agricultural Microbiology, Department of Crop Science, Agricultural University of Athens, 11855 Athens, Greece; georgioskoutrotsios@gmail.com (G.K.); giorgosbekiaris@yahoo.gr (G.B.); zervakis@aua.gr (G.I.Z.)

The authors wish to add the following information to the Authors and Affiliation section of our paper published in *Molecules* [1].

1. Change in authorship (change in full name of Mr. Christodoulou from Paraschos Christodoulou to Paris Christodoulou).
2. Addition of an extra affiliation to Mr. Paris Christodoulou as follows:

*Change from*

Affiliation 1: Institute of Chemical Biology, National Hellenic Research Foundation, 11634 Athens, Greece

*to*

Affiliation 1: Institute of Chemical Biology, National Hellenic Research Foundation, 11635 Athens, Greece

Affiliation 2: Department of Biochemistry and Biotechnology, University of Thessaly, 41500 Larissa, Greece
