# Supplementary material for: Genoprotective Properties and Metabolites of β-Glucan-Rich Edible Mushrooms Following Their In Vitro Fermentation by Human Faecal Microbiota
Source: Molecules. 2020 Aug 4;25(15):3554. doi: 10.3390/molecules25153554 (PMC7435999; doi:10.3390/molecules25153554)
Supplement: Supplementary file 1 [file molecules-25-03554-s001.pdf]

Table S1. Biological efficiency, productivity, and total, α- and β-glucans content of *Pleurotus ostreatus*, *P. eryngii*, *P. nebrodensis*, *P. citrinopileatus*, *Hericium erinaceus* and *Cyclocybe cylindracea* strains (WS: wheat straw; TPOMW: two-phase olive-mill waste; OLPR: olive leaves and prunings; GM: grape marc; OL: olive leaves). Strains appearing in bold typeface were selected for further study.

Species/Strain	Substrate	Biological Efficiency (%)	Productivity	Total glucans (%, d.w.)	α-glucans (%, d.w.)	β-glucans (%, d.w.)
***P. ostreatus* 1123**[1]	WS	77.3 ± 5.52	1.0 ± 0.2	39.4 ± 1.3	8.7 ± 0.4	30.6 ± 2.5
	WS:TPOMW 3:1	73.7 ± 1.1	1.0 ± 0.1	37.5 ± 0.6	6.0 ± 1.1	31.5 ± 0.4
	WS:TPOMW 1:1	71.3 ± 12.3	0.8 ± 0.1	31.7 ± 2.2	2.2 ± 0.6	29.6 ± 2.4
	WS:TPOMW 1:3	17.4 ± 8.2	0.2 ± 0.1	32.7 ± 1.4	4.7 ± 0.8	28.1 ± 1.5
	WS:OLPR 3:1	82.6 ± 14.5	1.1 ± 0.2	35.3 ± 1.9	7.2 ± 2.0	28.0 ± 1.6
	WS:OLPR 1:1	56.8 ± 6.2	0.7 ± 0.1	35.2 ± 3.4	6.3 ± 2.1	28.9 ± 3.5
	WS:OLPR 1:3	39.7 ± 1.4	0.5 ± 0.1	36.8 ± 1.7	5.7 ± 2.4	25.6 ± 0.2
	OLPR	44.7 ± 11.7	0.5 ± 0.2	38.5 ± 2.9	3.4 ± 0.6	35.1 ± 2.8
***P. ostreatus* LGM 22**[2]	WS	100.5 ± 15.0	3.1 ± 0.7	34.3 ± .2.1	6.6 ± 1.1	27.7 ± 2.4
	WS:GM 3:1	62.8 ± 3.2	1.9 ± 0.2	37.6 ± 4.7	4.0 ± 0.9	33.5 ± 4.4
	WS:GM 1:1	29.7 ± 8.0	0.9 ± 0.3	36.1 ± 2.4	6.7 ± 0.7	29.4 ± 2.5
	WS:GM 1:3	24.8 ± 5.6	0.6 ± 0.2	31.6 ± 0.7	5.1 ± 1.2	26.5 ± 0.6
	OL	29.3 ± 8.4	0.7 ± 0.3	35.5 ± 1.0	3.4 ± 0.7	32.1 ± 1.1
	OL:TPOMW 3:1	46.2 ± 13.7	0.7 ± 0.2	39.9 ± 0.5	4.9 ± 1.0	35.0 ± 0.4
	OL:TPOMW 1:1	55.4 ± 3.0	0.9 ± 0.0	34.3 ± 1.3	6.0 ± 0.8	28.3 ± 1.2
	OL:TPOMW 1:3	26.6 ± 13.0	0.6 ± 0.2	27.7 ± 3.9	3.5 ± 0.7	24.1 ± 3.6
P. ostreatus 104	WS	106.7 ± 7.8	1.6 ± 0.3	35.9 ± 1.1	6.2 ± 1.6	29.7 ± 1.0
	WS:GM 1:1	98.0 ± 19.6	1.8 ± 0.3	32.8 ± 3.5	5.6 ± 1.0	27.2 ± 3.4
	OL:TPOMW 1:1	27.0 ± 0.1	0.4 ± 0.1	24.4 ± 2.3	6.8 ± 0.5	17.6 ± 2.4
P. ostreatus CS	WS	79.7 ± 33.1	0.8 ± 0.2	29.4 ± 2.1	10.6 ± 2.0	18.8 ± 2.1
	WS:GM 1:1	84.1 ±22.5	1.9 ± 0.2	26.4 ± 0.7	6.8 ± 1.0	19.6 ± 0.6
	OL:TPOMW 1:1	17.9 ± 5.6	0.3 ± 0.2	28.8 ± 1.4	7.9 ± 3.0	20.9 ± 1.2
P. ostreatus 3046	WS	109.4 ± 17.9	3.7 ± 0.6	25.2 ± 2.1	2.2 ±0.3	22.9 ± 1.7
P. ostreatus P80	WS	69.8 ± 28.0	2.4 ± 0.9	19.5 ± 1.7	1.7 ±0.1	17.8 ±1.5
P. ostreatus P15[3]	WS	87.6 ± 8.4	2.9 ± 0.3	24.0 ± 4.2	2.3 ± 0.4	21.7 ± 1.8
P. ostreatus P57[3]	WS	90.6 ± 24.6	3.4 ± 1.0	19.4 ± 0.8	4.1 ± 0.5	15.3 ± 1.0
P. ostreatus P59[3]	WS	65.0 ± 12.1	2.4 ± 0.7	15.0 ± 0.5	4.0 ±0.3	10.9 ± 0.0
P. ostreatus P69[3]	WS	79.2 ± 16.2	2.0 ± 1.0	19.1 ± 0.7	3.8 ± 1.9	15.3 ± 0.5
P. ostreatus P112[3]	WS	97.2 ± 19.7	3.2 ± 1.1	14.5 ± 1.0	2.9 ± 0.2	11.5 ± 0.8
P. ostreatus P146[3]	WS	87.1 ± 18.0	4.2 ± 1.5	15.7 ± 0.3	4.1 ± 1.0	11.6 ± 0.3
P. ostreatus P149[3]	WS	87.0 ± 23.4	3.3 ± 1.2	19.2 ± 0.1	5.0 ± 0.8	14.2 ± 0.1
P. ostreatus P179[3]	WS	81.8 ± 21.1	4.3 ± 1.7	17.3 ± 0.1	4.5 ± 0.7	12.7 ± 0.7
P. ostreatus P182[3]	WS	85.1 ± 15.7	3.2 ± 0.6	21.5 ± 0.3	4.8 ± 1.1	16.7 ± 0.2
P. ostreatus LGAM 443[3]	WS	43.9 ± 9.3	1.5 ± 0.4	22.1 ± 0.8	5.2 ± 0.6	16.9 ± 0.4
***P. eryngii* LGAM 216**[2]	WS	57.3 ± 9.6	0.8 ± 0.1	46.6 ± 4.9	7.9 ± 3.3	38.7 ± 5.4

	WS:GM 1:1	87.2 ± 8.9	1.4 ± 0.2	49.7 ±1.3	7.6 ± 2.4	42.2 ±1.3
	OL:TPOMW 1:1	73.3 ± 8.8	1.1 ± 0.1	40.2 ±1.9	7.4 ± 1.5	32.8 ± 2.3
P. eryngii LGAM 170	WS	38.4 ± 3.1	0.4 ± 0.0	24.3 ±1.2	4.5 ± 1.0	19.8 ± 1.3
	WS:GM 1:1	48.2 ± 14.7	0.6 ± 0.0	28.0 ± 0.5	2.7 ± 0.1	25.3 ± 0.7
	OL:TPOMW 1:1	42.3 ± 14.8	0.6 ± 0.2	27.1 ± 0.9	2.7 ± 0.5	24.4 ± 3.2
P. nebrodensis UPA 6[2]	WS	35.9 ± 13.8	1.5 ± 0.4	28.6 ± 2.4	8.0 ± 1.3	20.6 ± 2.6
	WS:GM 3:1	28.3 ± 13.9	0.8 ± 0.4	35.5 ± 3.1	8.6 ± 1.4	27.0 ± 2.8
	WS:GM 1:1	36.8 ± 30.3	1.1 ± 0.4	32.6 ± 2.3	7.8 ± 1.5	24.8 ± 2.3
	WS:GM 1:3	37.6 ± 1.8	0.8 ± 0.2	37.7 ± 3.9	9.4 ± 1.8	28.3 ± 4.1
	OL	34.6 ± 4.6	0.2 ± 0.0	30.8 ± 1.0	8.0 ± 2.1	22.8 ± 0.8
	OL:TPOMW 3:1	11.9 ± 0.3	1.1 ± 0.3	29.6 ± 3.8	6.9 ± 2.0	22.6 ± 4.0
	OL:TPOMW 1:1	18.4 ±12.0	1.1 ± 0.1	35.7 ± 3.9	10.8 ± 2.4	24.9 ± 4.2
P. citrinopileatus	WS	53.7 ± 16.5	0.8 ± 0.3	30.3 ± 1.2	8.3 ± 2.5	22.0 ±1.1
	WS:GM 1:1	78.5 ± 1.7	1.5 ± 0.1	28.4 ± 1.1	5.7 ± 1.2	22.7 ± 1.2
	OL:TPOMW 1:1	26.2 ± 3.6	0.5 ± 0.1	27.2 ± 0.4	6.7 ± 2.4	20.5 ± 0.1
H. erinaceus[4]	BS	6.0 ± 0.2	0.1 ± 0.0	16.4 ± 0.1	1.0 ± 0.1	15.4 ± 0.2
	BS:OLPR 3:1	6.4 ± 0.1	0.1 ± 0.0	16.8 ± 0.7	0.9 ± 0.1	15.8 ± 0.6
	BS:OLPR 1:3	24.3 ± 0.5	0.3 ± 0.0	16.6 ± 0.1	0.9 ± 0.1	15.7 ± 0.1
	OLPR	30.8 ± 2.7	0.3 ± 0.0	21.8 ± 0.2	1.1 ± 0.1	20.7 ± 0.3
***C. cylindracea* CC2**	WS	109.7 ± 14.4	1.2 ± 0.1	39.3 ± 2.1	6.1 ± 0.5	33.2 ± 1.9
	WS:OLPR 1:3	69.8 ± 6.9	0.7 ± 0.0	35.0 ± 5.4	8.6 ± 1.1	26.3 ± 4.8
C. cylindracea CC3	WS	118.3 ± 19.9	1.3 ± 0.3	35.5 ± 3.2	8.2 ±0.4	27.2 ± 3.00
	WS:OLPR 1:3	30.2 ± 13.8	0.3 ± 0.1	36.4 ± 1.9	7.1 ± 0.2	29.3 ± 2.0
C. cylindracea CC4	WS	60.0 ± 4.7	0.7 ± 0.2	32.0 ± 4.7	5.1 ± 0.7	26.8 ± 3.9
C. cylindracea 4022	WS	59.2 ±1.3	0.8 ± 0.1	31.0 ± 1.8	2.5 ± 0.8	28.5 ± 2.2
C. cylindracea 480	WS	53.9 ± 5.8	0.8 ± 0.0	36.2 ± 3.7	5.1 ± 1.5	31.1 ± 3.8
C. cylindracea 493	WS	73.9 ± 9.1	1.0 ± 0.1	41.6 ± 6.9	8.2 ± 0.5	33.4 ± 6.8
***C. cylindracea* 505**	WS	48.3 ± 3.3	0.6 ± 0.0	40.6 ± 4.5	3.4 ± 1.1	37.2 ± 3.8
C. cylindracea 515	WS	61.8 ± 4.1	0.8 ± 0.1	34.0 ± 2.7	6.7 ± 2.6	27.3 ± 3.0

[1,2,3,4] values from Koutrotsios et al. (2019), Koutrotsios et al. (2018), Koutrotsios et al. (2017) and Koutrotsios et al. (2016), respectively.

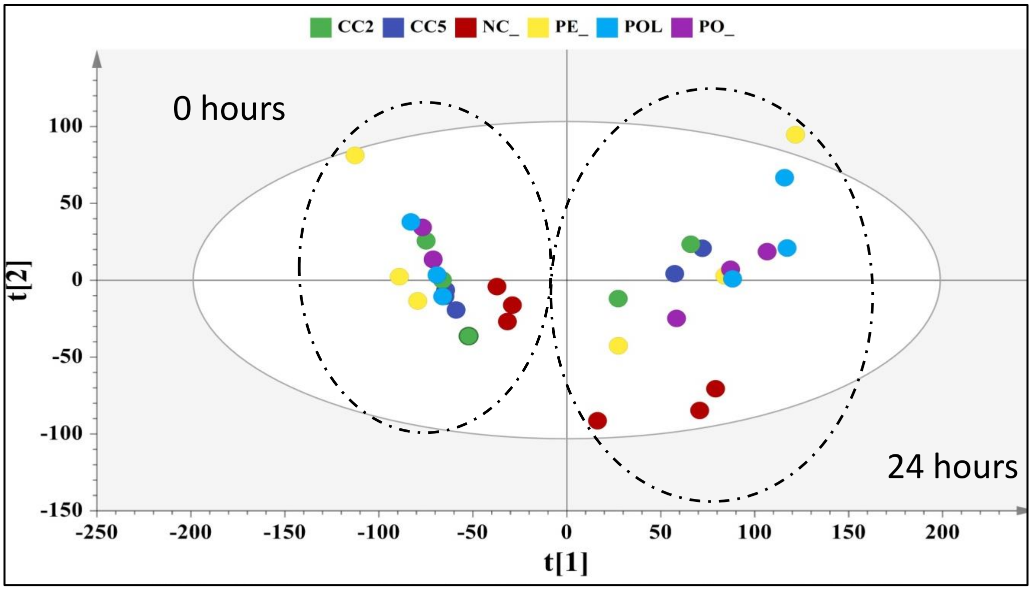

Figure S1. PCA analysis of the NMR data for the pre- and post-fermentation supernatants. (R^2X(cum)=0.70, Q^2(cum)=0.60, Pareto scaling, Hotelling T^2=95%). Fecal inocula derived from 3 donors. PO; *P. ostreatus* strain 1123, POL; *P. ostreatus* strain LGM 22, PE; *P. eryngii* strain LGAM 216, CC2; *C. cylindracea* strain 2, CC5; *C. cylindracea* strain 505. NC; basal medium with no additional carbohydrate source.
